# Prodigiosin/PU-H71 as a novel potential combined therapy for triple negative breast cancer (TNBC): preclinical insights

**DOI:** 10.1038/s41598-020-71157-w

**Published:** 2020-09-07

**Authors:** Mohammed Moustapha Anwar, Manal Shalaby, Amira M. Embaby, Hesham Saeed, Mona M. Agwa, Ahmed Hussein

**Affiliations:** 1 Department of Biotechnology, Institute of Graduate Studies and Research, Alexandria University, Alexandria, Egypt; 2 Medical Biotechnology Department, Institute of Genetic Engineering, City of Scientific Research and Technological Applications, Alexandria, Egypt; 3 Department of Chemistry of Natural and Microbial Products, Pharmaceutical and Drug Industries Research Division, National Research Centre, 33 El-Behooth St, Dokki, Giza 12311, Egypt

**Keywords:** Biotechnology, Cancer

## Abstract

Prodigiosin, a secondary metabolite red pigment produced by *Serratia marcescens*, has an interesting apoptotic efficacy against cancer cell lines with low or no toxicity on normal cells. HSP90α is known as a crucial and multimodal target in the treatment of TNBC. Our research attempts to assess the therapeutic potential of prodigiosin/PU-H71 combination on MDA-MB-231 cell line. The transcription and protein expression levels of different signalling pathways were assessed. Treatment of TNBC cells with both drugs resulted in a decrease of the number of adherent cells with apoptotic effects. Prodigiosin/PU-H71 combination increased the levels of caspases 3,8 and 9 and decreased the levels of mTOR expression. Additionally, there was a remarkable decrease of HSP90α transcription and expression levels upon treatment with combined therapy. Also, EGFR and VEGF expression levels decreased. This is the first study to show that prodigiosin/PU-H71 combination had potent cytotoxicity on MDA-MB-231 cells; proving to play a paramount role in interfering with key signalling pathways in TNBC. Interestingly, prodigiosin might be a potential anticancer agent to increase the sensitivity of TNBC cells to apoptosis. This study provides a new basis for upcoming studies to overcome drug resistance in TNBC cells.

## Introduction

Breast cancers represent a collection of malignancies that arise either in the breast tissue made up of glands for milk production, called lobules or in the ducts connecting the lobules to the nipple^1,2^. More than 60% of breast cancers are either estrogen receptor (ER) or progesterone receptor (PR) positive. Other defined subtypes are characterised by human epidermal growth factor receptor 2 (HER2) protein overexpression or HER2 gene amplification, and triple negative breast cancer (TNBC), where there is neither ER/PR expression nor HER2 overexpression^2,3^. Up to 20% of breast cancers are TNBCs, occurring often in women ˂ 50 years of age^4^. Despite the profound increase in breast cancer awareness and management, it is the 5th leading cause of death in women with dramatically increased rates in almost every region around the world^5–8^. According to the world health organisation (WHO), breast cancer was expected to comprise nearly 15% of all cancer-related mortality in 2018^9^. The global burden of cancer (GLOBOCAN) statistics 2018 demonstrated that it is the most commonly diagnosed cancer and the leading cause of cancer-related deaths among females^10^. It is noteworthy that substantial evolution in our comprehension of the disease has led to notable advancements in the early prevention, detection, and treatment. The clinical focus is currently heading towards tailored therapy to characterise more targets with highly unprecedented approaches^11^. A number of investigational therapies such as heat shock protein 90 alpha (HSP90α) inhibitors, autophagy inducers, and PI3K-AKT-mTOR pathway inhibitors could hold promise^12,13^.

Evolving research shows that natural molecules are vital for targeting crucial cancer hallmarks^13^. In this regard, it has been shown that prodigiosin (2-methyl-3-pentyl-6-methoxyprodigiosin), a secondary metabolite red pigment produced by the Gram-negative bacilli *Serratia marcescens*, has interesting apoptotic effects on a diverse array of human cancer cells with multidrug resistant phenotypes or defects in apoptotic pathways, with low or no toxicity in normal cells^13–16^. Prodigiosin synthesis is mediated by Quorum Sensing (QS) activity (the regulation of gene expression in response to fluctuations in cell-population density to produce and release autoinducers that increase in concentration as a function of cell density). Cyclic-adenosine-monophosphate (cAMP) receptor protein and cyclic-di-guanosine-monophosphate (cGMP), pH, and temperature via regulating the expression of the prodigiosin biosynthetic genes (pigA-N) help in synthesis as well^15^. Prodigiosin is considered as the prototype of bacterial prodiginines, a family of tripyrrole red compounds, which is characterised by a common pyrrolyl dipyrromethene skeleton with 4-methoxy, 2–2 bipyrrole ring system^15^. Generally, bacterial prodiginines have been classified into linear and cyclic derivatives; prodigiosin and undecylprodigiosin, and streptorubin B, cycloprodigiosin, and cyclononylprodigiosin, respectively. Prodigiosin usually exists in two interconverting rotamers, Cis (or β) and Trans (or α), which are controlled by the pH of the solution^17,18^. In addition to its anticancer activity, prodigiosin was found to exhibit antibacterial, antifungal, antiprotozoal, antimalarial, and immunosuppressive properties^14–19^. Prodigiosin exhibits anticancer effects due to its proapoptotic action regardless of p53 status. Recently, proapoptotic effects in cancer cells via regulation of apoptotic and antiapoptotic genes have been attributed to prodigiosin^20^.

Heat shock proteins are a unique family of proteins, most famous is their role as molecular chaperones with highly conserved cytoprotective structures^21,22^. They are characterised by their overexpression in cells that are exposed to stressful circumstances as a line of defence mechanism against the outer environment (such as exercise, gravity, heat, oxygen), protecting them during proteotoxic stress. As a common mechanism of action, HSPs are involved in various important cellular processes such as multiprotein assembly, secretion, trafficking, protein degradation, receptor maturation, signal transduction and regulation of transcription factors^21,22^. Heat shock protein 90 is a small family of 80–90 KDa chaperones, highly conserved across species and almost ubiquitously expressed, and is considered as the key regulator of proteostasis^23,24^. It is regarded as an ATP-dependent molecular chaperone, playing a key role in stabilising and activating > 200 client proteins. It is divided into HSP90α (encoded by *HSP90AA1*) and HSP90β (encoded by *HSP90AB1*)^23–25^. HSP90 family is recognised as a suitable target for cancer therapy and is expected to actively elaborate in tumour cell proliferation, metastatic invasion, and death^21–25^.

Several HSP90 inhibitors are designed to target either the N-terminus or the C-terminus of the protein. For example, retaspimycin hydrochloride (IPI-504), a derivative of geldanamycin and 17-AAG, is proven effective in glioma cell lines^26,27^. In the preclinical setting, the inhibition of HSP90 could be effective against resistant tumours, such as mutant EGFR-driven lung adenocarcinoma^25,28^.

Benzoquinone ansamycin and radicicol (RD) analogues are HSP90α inhibitors with the advantage of the folded structure endorsed by the binding of GA and RD to HSP90α. PU3, the first inhibitor that showed efficacy in cancer cell lines was not as effective as 17-AAG. Further analysis of PU3 and its interaction with HSP90α resulted in the synthesis of PU-H71 “6-Amino-8-[(6-iodo-1,3-benzodioxol-5-yl)thio]-*N*-(1-methylethyl)-9*H*-purine-9-propanamine”. Interestingly, PU-H71 is only required in minute concentrations to inhibit HSP90α with a higher affinity towards tumourigenic cells^25,29^. PU-H71 is a newer water-soluble purine-analogue and is considered the most promising HSP90 inhibitor, having potent selectivity for HSP90 in epichaperome networks^30^. Presently, it is being tested in phase I clinical trials (NCT01393509 and NCT01581541) for patients with advanced solid malignancies^29–31^.

Combination treatment has given the most successful anticancer outcomes with superior predominance to target different pathways to minimise treatment resistance^32^. Combining distinctive particles utilised to target different markers may be an ideal route^33^. Cell lines are extensively used models to consider metastatic cancer, where MDA-MB-231 cells are the most suitable cell line to study metastatic breast cancer^34^. At present, combining diverse compounds gained attraction in nearly 80% of clinical trials for interesting results in TNBC patients after failure of the single treatment^35,36^. Regardless the gigantic endeavours concerted on combination therapy, there are a few special cases such as bevacizumab/chemotherapy combination that showed no clinical benefit^37^. To the best of knowledge, this study is the first attempt to investigate the therapeutic potential of combining prodigiosin to PU-H71 on MDA-MB-231 cells.

## Results

### Production, extraction, and purification of prodigiosin

**Figure 1:**
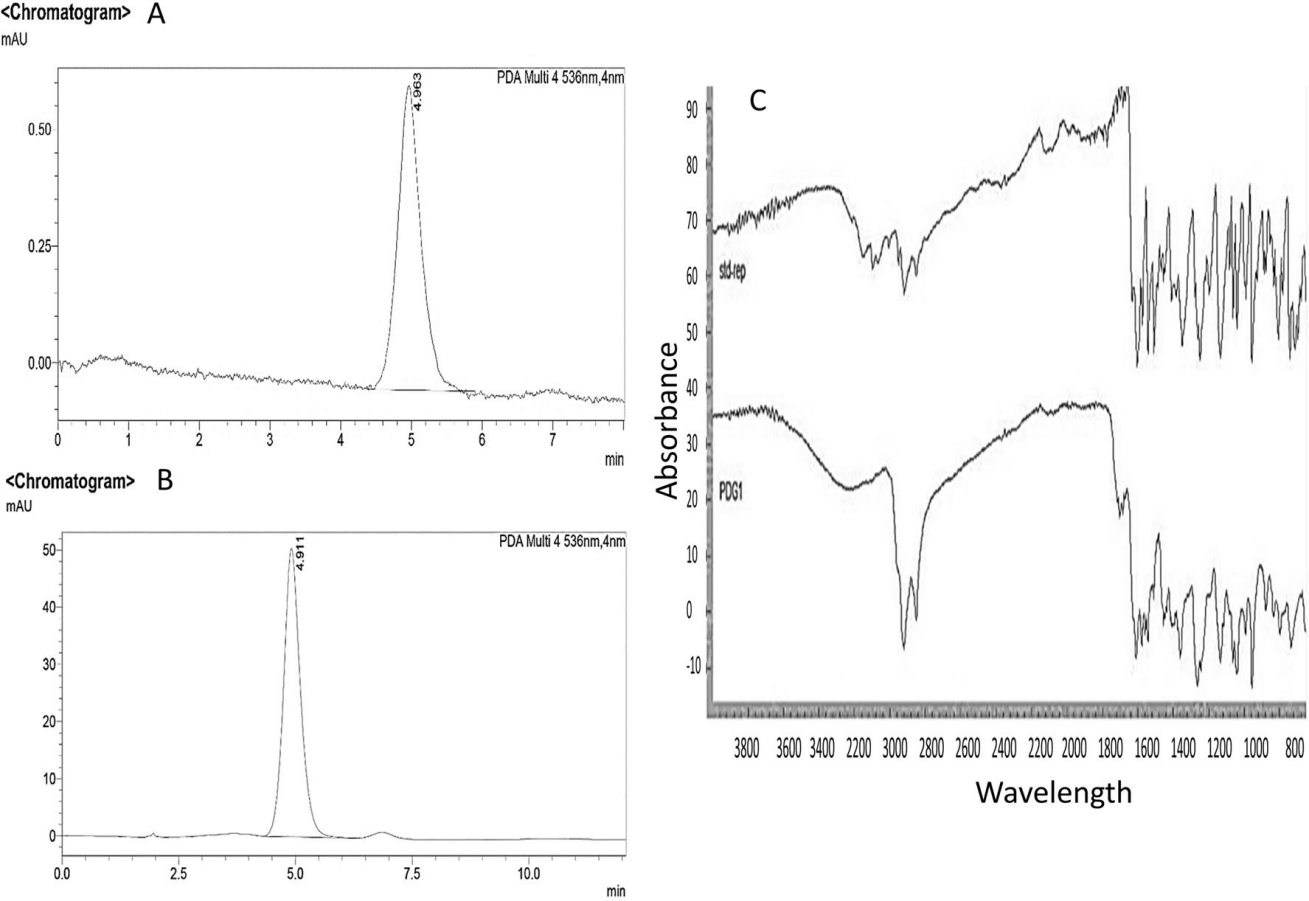
HPLC profile of standard prodigiosin **(a)**, HPLC profile of *S. marcescens* prodigiosin obtained from silica gel fraction **(b)**, FTIR spectrum of the purified silica gel *S. marcescens* prodigiosin fraction as compared to the standard prodigiosin **(c)**. *STD* standard prodigiosin, *PDG1* *S. marcescens* prodigiosin.

Large amounts of prodigiosin (7 L) were obtained after 24 h upon using the laboratory scale fermenter and were further purified as described in materials and methods section. The purified extract (0.99 g) was subjected to characterisation using high-performance liquid chromatography (HPLC) profile and Fourier-Transform Infrared (FTIR) analyses. Both results were in consistent with a previous study published by Patil and his group^38^. These findings revealed that the purified *S. marcescens* prodigiosin was of appropriate purity and quality; almost quite similar to that of the standard pigment (Fig. 1).

### The neutral red viability assay

**Figure 2:**
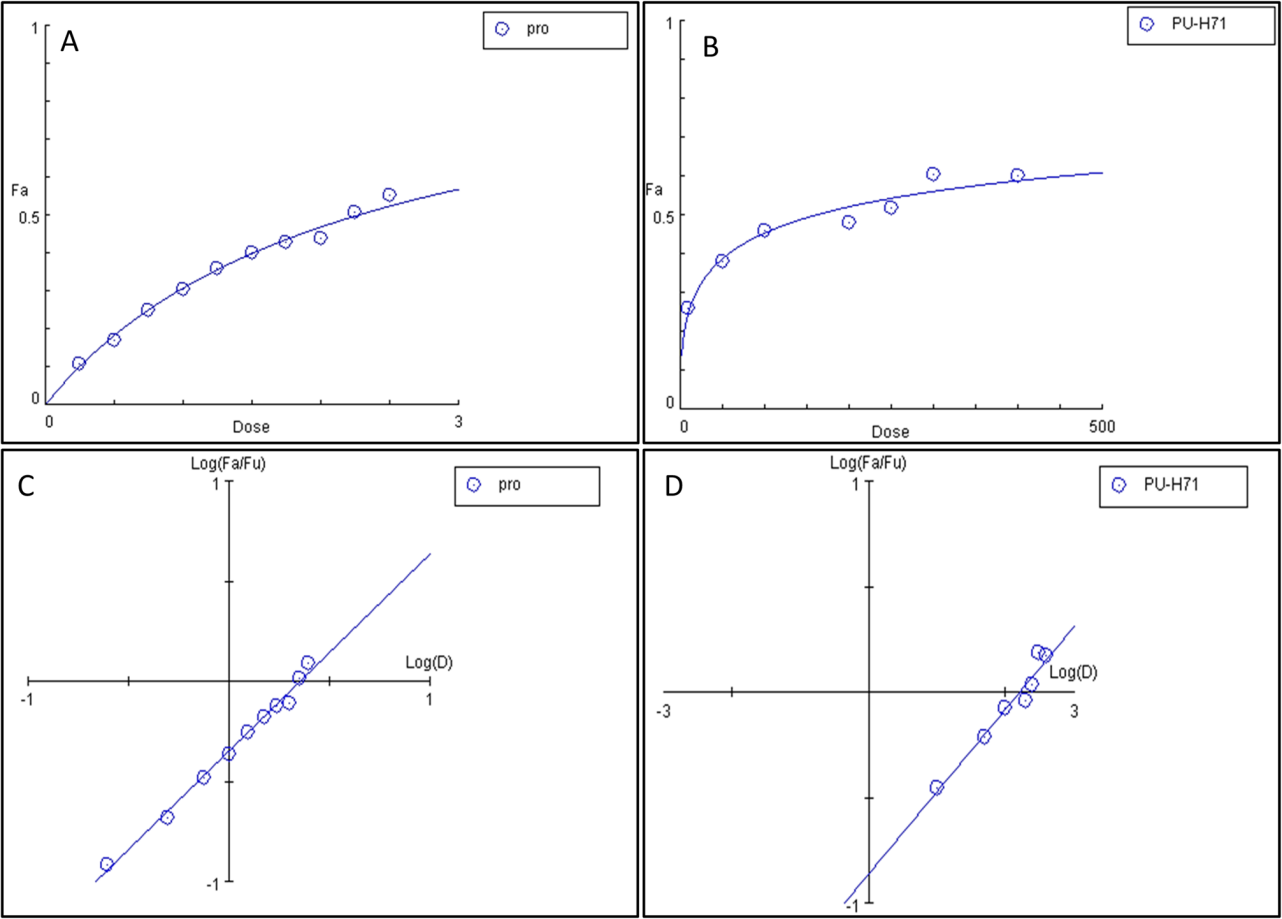
Dose–response curve or prodigiosin **(a)**, Dose–response curve for PU-H71 **(b)**, Median-effect curve for prodigiosin **(c)**, Median-effect curve for PU-H71 **(d)**, *Fa* affected fractions of treated cells, *Fu* unaffected fractions of treated cells, *Pro* prodigiosin.

Percentage of inhibition and IC_50_ values of both prodigiosin and PU-H71 via the neutral red assay in a 96-well tissue culture plate were calculated. It was shown that the percentage of viable cells proportionally decreased with increasing the doses of both prodigiosin and PU-H71 (Tables 1 and 2). At 540 nm, linear relationships have been observed between the doses of prodigiosin, PU-H71, and the affected fractions of MDA-MB-231 cells (Fig. 2). After 48 h, the dose–response curves of prodigiosin and PU-H71 using COMPUSYN software, showed a median inhibitory concentration of 2.1 µM (r = 0.99) and 157.88 nM (r = 0.98), respectively. The linear correlation coefficient (r) of the median-effect plots signifies the conformity of the current data to the mass-action law for in-vitro experiments. The cytotoxic effects were directly proportional to the dose of prodgiosin and PU-H71. Of note, there was no statistically significant difference between the results of both the negative and the vehicle-treated control groups in all the studied parameters (*P* > 0.05).

**Table 1:** Percentage of inhibition and IC_50_ values of prodigiosin by the neutral red viability assay.

Drug concentration (µM)	Untreated cells	0.25	0.50	0.75	1.00	1.25	1.50	1.75	2.00	2.25	2.50
Mean absorbance at 540 nm	0.26	0.23	0.22	0.19	0.18	0.17	0.15	0.15	0.14	0.13	0.11
Percentage inhibition (%)	NA (non-applicable)	10.90	17.20	25.00	30.40	36.00	40.10	43.00	44.00	51.0	55.20
Determined IC_50_ (µM)	2.1

**Table 2:** Percentage of inhibition and IC_50_ values of PU-H71 by the neutral red viability assay.

Drug concentration (nM)	Untreated cells	10	50	100	200	250	300	400
Mean absorbance at 540 nm	1.832	1.430	1.185	1.033	0.943	0.875	0.679	0.637
Percentage inhibition (%)	NA (non-applicable)	21.9	35.3	43.6	48.5	52.2	62.9	65.2
Determined IC_50_ (nM)	157.88

**Figure 3:**
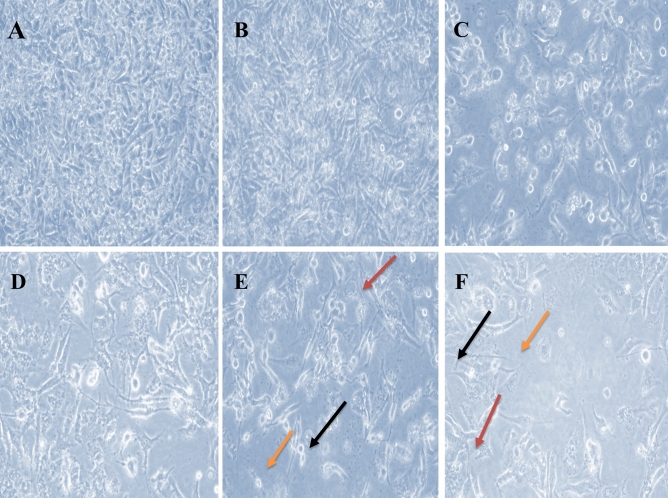
Anticancer activity using the IC_50_ of prodigiosin, PU-H71, and the effective dose combination on MDA-MB-231 breast cancer cell line by the neutral red viability assay. Untreated cells **(a)**, DMSO-treated cells **(b)**, 2.1 µM **(c)**, 157.88 nM **(d)**, combination of prodigiosin (1.13 µM) and PU-H71 (78.94 nM) **(e, f)**. Black arrows refer to the rounded floating cells, orange arrows refer to the absence of close contact spindle-shape adherent cells, and red arrows refer to the cell shrinkage without microvilli.

Based on the % inhibition, the calculated IC_50_ for prodigiosin (2.1 µM) and PU-H71 (157.88 nM) were proved to kill 50% of the MDA-MB-231 cells (Fig. 3). Furthermore, a combination of half of the IC_50_ values of both drugs showed the maximum % inhibition (75.14%) (Fig. 3) as compared to other doses of the combinations. Interestingly, combination index (CI) analysis revealed that such dose was synergistic (CI = 0.7) in comparison with different doses of the drugs’ combinations^39,40^.

### The effect of prodigiosin/PU-H71 combination on the viability and morphology of MDA-MB-231 cells

Treatment with different doses of prodigiosin, PU-H71 and their combination resulted in a more spherical appearance and an increase in the number of the floating cells accompanied with a marked decrease in the number of live (adherent) cells in comparison with the untreated (normal) and Dimethyl Sulfoxide (DMSO)-treated cells (Fig. 3).

### The effect of treatment on *BAX* transcription level

**Figure 4:**
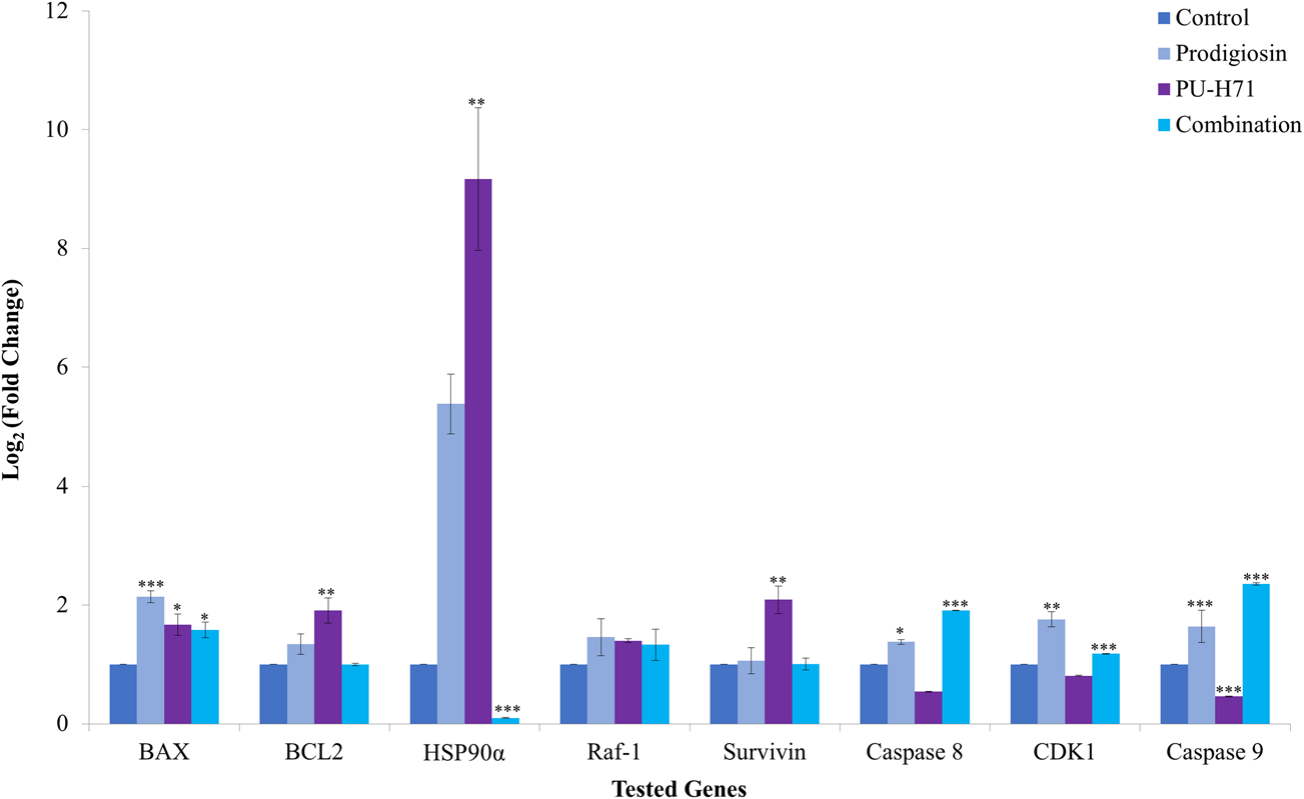
The effect (Log_2_, fold change) of prodigiosin, PU-H71, and their combination on *BAX*, *BCL2*, *HSP90α*, *Raf-1*, *Survivin*, *Caspase 8*, *CDK1*, and *Caspase 9* transcription levels using Real time PCR. *Significant difference against control (*P* < 0.05), **Very significant difference against control (*P* < 0.01), ***Extremely significant difference against control (*P* < 0.001).

*BAX* gene transcription could provide clearer insights about the extent of MDA-MB-231 cells' resistance to prodigiosin, PU-H71 or their combination. Prodigiosin produced a statistically significant upregulation in the mRNA transcription of *BAX* amongst all treatment groups with 2.14-fold the control value (*P* < 0.001). Similarly, treatment with either PU-H71 or the combined therapy significantly increased the mRNA transcription of *BAX* to 1.67-fold and 1.58-fold the control value, respectively (*P* < 0.05) (Fig. 4).

### The effect of treatment on *BCL2* transcription level

Since prodigiosin and PU-H71 were reported to downregulate the level of *BCL2* gene transcription in breast cancer cells^41,42^, and since TNBC is refractory to current treatment, to this end, we determined whether PU-H71, prodigiosin and their combination could inhibit *BCL2* in MDA-MB-231 cell line. In this context, BCL2 dysregulation promotes innate or acquired treatment resistance and contributes to evade apoptosis, a hallmark of cancer^43^. The results presented inferred that treatment with prodigiosin/PU-H71 combination had no effect on the mRNA transcription of *BCL2* as compared to the control group in the MDA-MB-231 cell line (Fig. 4). On the contrary, PU-H71 alone caused a statistically significant increase in the mRNA transcription to 1.91-fold in comparison with the control group (*P* < 0.01). The mRNA transcription upon treatment with prodigiosin alone increased but in a nonsignificant behaviour.

### Effect of treatment on HSP90α transcription and expression levels

**Figure 5:**
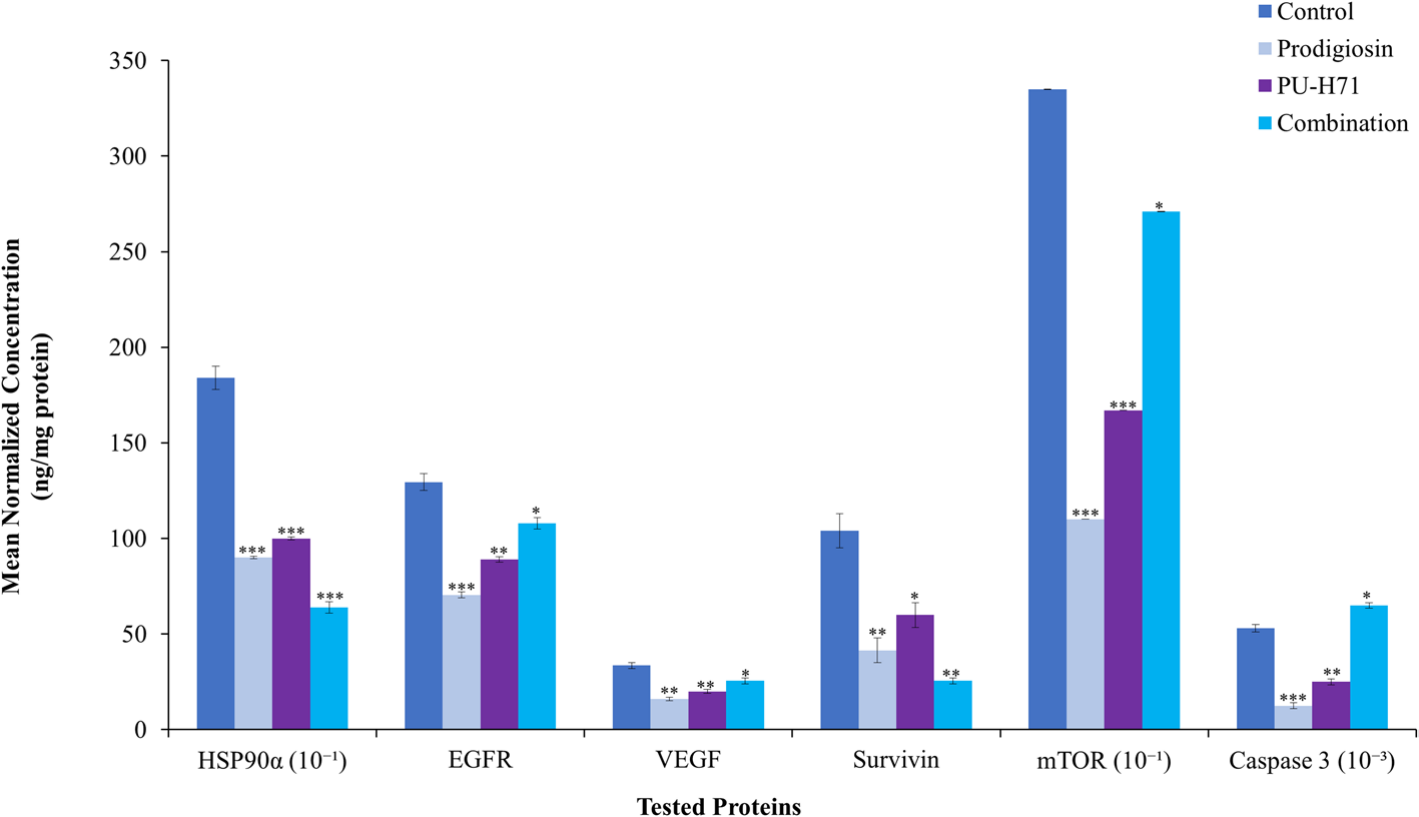
The effect (mean normalized concentration, ng/mg protein) of prodigiosin, PU-H71, and their combination on HSP90α, EGFR, VEGF, Survivin, mTOR and Caspase 3 expression levels using ELISA. *Significant difference against control (*P* < 0.05), **Very significant difference against control (*P* < 0.01), ***Extremely significant difference against control (*P* < 0.001).

Treatment with prodigiosin/PU-H71 combination showed a nonsignificant decrease in the *HSP90α* transcription, reaching 0.1-fold the control values (*P* > 0.05). However, it exhibited a statistically significant reduction in *HSP90α* mRNA transcription levels as compared to the PU-H71- and prodigiosin-treated groups (*P* < 0.001, *P* < 0.01, respectively). The PU-H71-treated group had a statistically significant increase in the mRNA transcription of *HSP90α* to about 9.00-fold control value (*P* < 0.001) (Fig. 4). Prodigiosin alone had a statistically significant increase in the mRNA transcription of *HSP90α* to 5.38-fold that of the control value (*P* < 0.01). Protein expression level of HSP90α was also examined using ELISA demonstrated that the combined therapy significantly downregulated the level of HSP90α to 0.64-fold the control value (*P* < 0.001) (Fig. 5).

### Effect of treatment on survivin transcription and expression levels

The application of PU-H71 showed a statistically significant increase in the *survivin* transcription up to 2.09-fold the control value (*P* < 0.01) in comparison with prodigiosin that had no effect on *survivin* transcription level. Combined therapy on the other hand decreased the transcription level of *survivin* to 1.01-fold the control value in a nonsignificant behaviour (*P* > 0.05) (Fig. 4). Using ELISA, prodigiosin, PU-H71 and their combination significantly downregulated the level of survivin protein expression when normalised to total protein level by 60% (*P* < 0.01), 42% (*P* < 0.05), and 75% (*P* < 0.01), respectively in comparison with the control group (Fig. 5).

### Effect of treatment on *Raf-1* transcription level

There was no observed significance on the level of *Raf-1* transcription upon treatment with either prodigiosin, PU-H71 or their combination (Fig. 4).

### Effect of treatment on *CDK1* transcription level

Amongst all treatment groups, prodigiosin produced a significant increase in the *CDK1* transcription level to 1.76-fold the control values (*P* < 0.01). Also, the combination therapy increased the transcription level of *CDK1* to 1.18-fold the control values (*P* > 0.05), but it was nonstatistically significant (*P* > 0.05) (Fig. 4). PU-H71 alone displayed a significant decrease (*P* < 0.01) in the *CDK1* transcription was observed as compared to prodigiosin-treated group^31^.

### Effect of treatment on *caspase 9* transcription level

Treatment with PU-H71/prodigiosin combination had a statistically significant increase in *caspase 9* transcription up to 1.91-fold the control values (*P* < 0.001). On the contrary, the PU-H71-treated group significantly decreased the transcription of *caspase 9* in comparison with all groups (0.54-fold the control values) (*P* < 0.001). Prodigiosin alone significantly increased *caspase 9* transcription to 1.38-fold the control values (*P* < 0.05) (Fig. 4).

### Effect of treatment on *caspase 8* transcription level

In this study, a combination of prodigiosin/PU-H71 significantly increased *caspase 8* transcription to 2.36-fold the control values (*P* < 0.001). On the other hand, PU-H71 decreased the levels of *caspase 8* transcription, which was nonsignificant (0.46-fold) (*P* > 0.05). The prodigiosin-treated group showed a significant increase of *caspase 8* transcription to 1.64-fold the control values (*P* < 0.05) (Fig. 4).

### Effect of treatment on EGFR expression level

Using ELISA, prodigiosin, PU-H71, and their combination significantly reduced the levels of EGFR protein expression by 16% (*P* < 0.001), 31% (*P* < 0.01), and 45% (*P* < 0.05), respectively (Fig. 5).

### Effect of treatment on VEGF expression level

In the prodigiosin-treated group, the levels of the VEGF protein decreased by 52% (*P* < 0.01) as compared to control. PU-H71 reduced VEGF level by 40% while combination treatment significantly decreased the level of VEGF protein expression by approximately 24% in comparison with the control group (Fig. 5).

### Effect of treatment on mTOR expression level

All treatments decreased the expression levels of mTOR with a varied degree of significance. PU-H71- and prodigiosin-treatments resulted in a statistically significant reduction to approximately 1.6- and 1.00-fold the control values, respectively (*P* < 0.001). Nevertheless, the combined therapy increased the expression levels when compared with prodigiosin-treated group alone (*P* < 0.001), but a significant decrease to almost 2.7-fold the control value (*P* < 0.05) (Fig. 5).

### Effect of treatment on caspase 3 expression level

Prodigiosin/PU-H71 combination resulted in a statistically significant upregulation of the level of the caspase 3 protein (Fig. 5) when compared to the prodigiosin- and PU-H71-treated groups (*P* < 0.001). This increase was also significant when compared to the control group by almost 23% (*P* < 0.05). Prodigiosin and PU-H71 had a significant downregulation of the level of caspase 3 protein by 76% (*P* < 0.001) and 53% (*P* < 0.01), respectively as compared to control.

### In-silico absorption, distribution, metabolism, and excretion (ADME) prediction of prodigiosin and PU-H71

**Figure 6:**
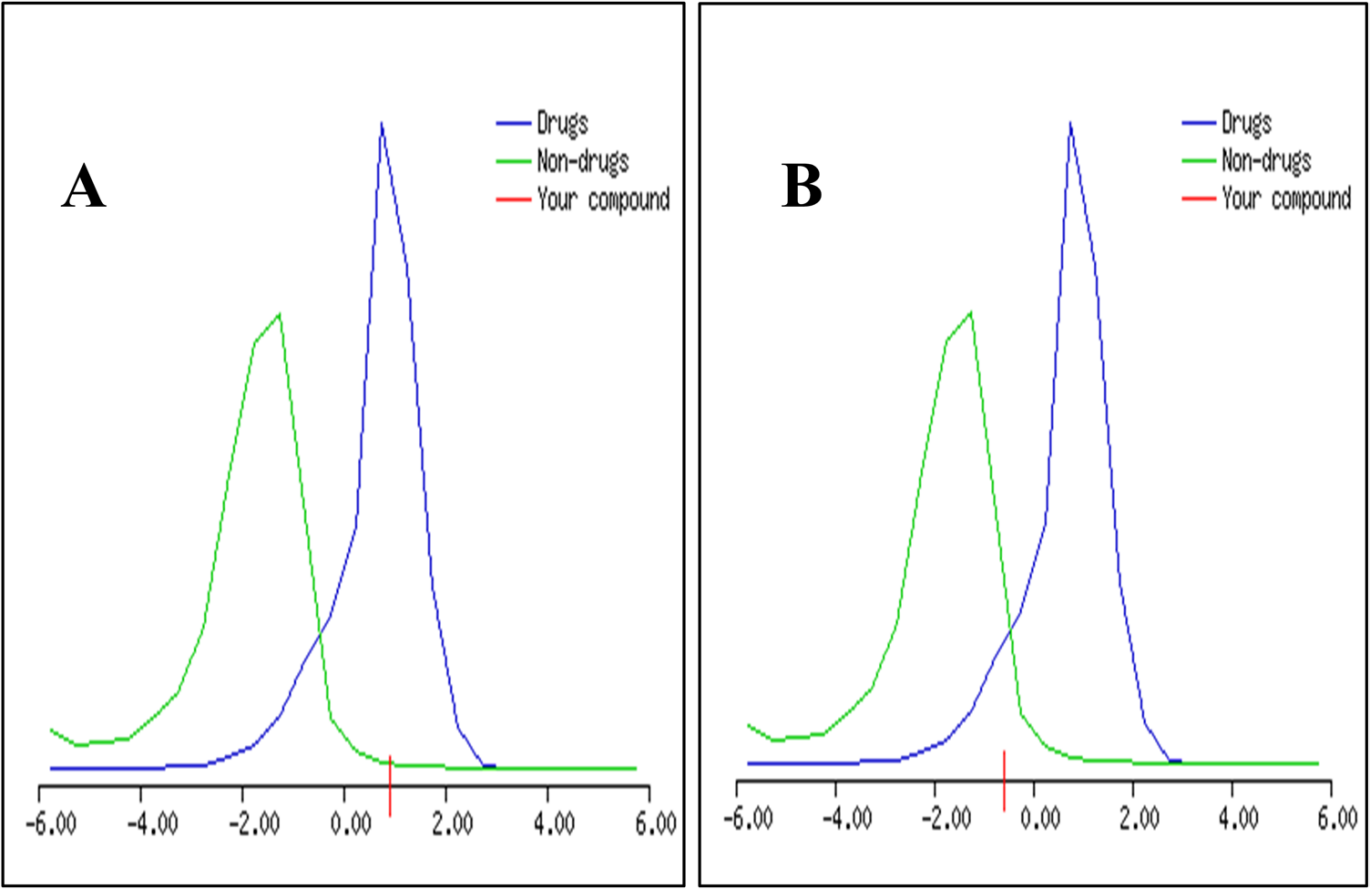
The MOLSOFT drug-likeness model of PU-H71 **(a)**, The MOLSOFT drug-likeness model of prodigiosin **(b)**.

Using SWISSADME (Table 3)^44^, a free web tool to evaluate the pharmacokinetics and medicinal chemistry friendliness of small molecules. The gastrointestinal absorption of PU-H71 and prodigiosin was high and both drugs exhibited moderate water solubility based on the Log S scale. According to the globally harmonised system of classification of labelling chemicals (GHS), prodigiosin and PU-H71 were shown to belong to classes V (LD_50_ mg/kg = 5,000) and III (LD_50_ mg/kg = 230), respectively^45^. Via MOLSOFT toolkit^46^, PU-H71 and prodigiosin were predicted to have drug-likeness scores of 0.93 and − 0.57, respectively (Fig. 6). Using SWISSADME^44^, prodigiosin has proved to be a good drug candidate with regards to the Ghose (Amgen), Veber (GlaxoSmithKline), Egan (Pharmacia), Muegge (Bayer) methods. On the other hand, PU-H71 has been confirmed as a drug-like molecule regarding the Veber, Egan, and Muegge filters only. With regards to synthetic accessibility (SA), prodigiosin and PU-H71 remarkably exhibited SA scores of 4.29 and 3.61, respectively^44^.

**Table 3:** Molecular properties and drug-likeness.

Drug name	Prodigiosin	PU-H71
Molecular formula	C_20_H_25_N_3_O	C_18_H_21_IN_6_O_2_S
Molecular weight	323.43	512.37
Log *P*_o/w_ (MLog P)	1.58	2.12
Number of hydrogen bond acceptors	2	6
Number of hydrogen bond donors	2	2
Topological polar surface area (TPSA)	53.17 Å^2^	125.41 Å^2^
Lipinski’s rule of 5	Yes (0 violation)	Yes (1 violation)

Prodigiosin has a molecular weight in the acceptable range (Mwt ≤ 500) in contrast to PU-H71. The MLogP (Octanol/water partition coefficient) of prodigiosin and PU-H71 were calculated and found to be within the acceptable range according to Lipinski’s rule (Table 3)^44^. As well, the Topological polar surface area (TPSA) for both drugs was shown to be in the optimal range for polarity^44^. Both drugs follow the Lipinski’s rule of five.

## Discussion

Due to the notable aggressive behaviour of TNBC, existing treatment options have limited or no efficacy against tumour metastasis^44,47^. Recently, Different molecular studies have allowed for the emergence of targeted therapies^48^, whereas others have evolved to explain mechanisms of resistance^49–53^. Several signalling pathways and biomarkers have been shown to be implicated in TNBC progression such as BAX/BCL2, HSP90α, EGFR, VEGF, survivin, CDK1, caspases, mTOR, and Ras/Raf/MEK/ERK^22,52–60^. MDA-MB-231 cells are the typical cell line used in cancer research as a tool to explore the mechanisms of numerous diseases, and identify new therapeutic targets^61^. Currently, various target molecules are either combined dependent on molecular functions of tumour markers or manifestations of additive or synergistic effects in cell lines^33^. In this aspect, the main focus of the current work was to explore the therapeutic role of a novel anticancer combination of PU-H71/prodigiosin on MDA-MB-231 TNBC cell line as an invasive phenotype in-vitro, as compared to their individual treatments.

The American National Cancer Institute (NCI) reported that a promising anticancer agent for future bioguided studies would have a significant cytotoxicity effect if it exerts an IC_50_ value ≤ 30 μg/mL. We demonstrate in this paper that prodigiosin induces apoptosis in MDA-MB-231 cells with an IC_50_ of 2.1 μM (0.73 μg/mL). Such dose was described to be sufficiently low to avoid any possible nonspecific effects^62^. Interestingly, according to the NCI, prodigiosin was also reported to have potent antineoplastic activity with the same IC_50_ against 57 different human cancer cell lines^63,64^. As well, the calculated dose of PU-H71 was reported to have antitumour activities in TNBC cell line models as reported before^31^. Phenotypically, with epithelial-like morphology, MDA-MB-231 cells develop as spindle-shaped cells, maintain their close contact, exhibit abundant microvilli, and cellular crowding with cytoplasmic connection, suggesting healthy proliferation (Fig. 3a). After 48 h under a phase-contrast inverted microscope, in all treatment groups (IC_50_ prodigiosin, IC_50_ PU-H71, and their combination), the MDA-MB-231 cells lost their elongated spindle-shape morphology associated with more suspension cells (dead cells). As well, morphologically damaged MDA-MB-231 cells become rounded and floating, were shrunk with the disappearance of microvilli (Fig. 3c–f).

The neutral red uptake assay is one of the most reliable cytotoxicity assays for the quantitative estimation of the number of viable cells in most primary cells and cell lines from diverse origin in different biomedical applications. Its principle is based on the ability of viable cells to integrate and bind the supravital dye neutral red in the lysosomes^39^. Using the neutral red viability assay, the effect of prodigiosin and PU-H71 has been shown to be dose-dependent. By increasing the dose, prodigiosin and PU-H71 individually have shown an observed proportional decrease in the MDA-MB-231 live cells’ count after 48 h (Tables 1 and 2), when compared to the untreated and DMSO-treated cells. Substantially, a combination of prodigiosin/PU-H71 (i.e. half IC_50_ of prodigiosin combined with half IC_50_ of PU-H71) has resulted in a maximum inhibition of approximately 75% (Fig. 3e,f) amongst other doses of prodigiosin/PU-H71 combinations. Of great importance, this dose of two-drug combination has shown a synergistic effect as demonstrated by the CI method (CI = 0.7) upon using COMPUSYN analysis that allows the quantitative determination of drug interactions, where CI = 1, < 1 and > 1 denotes additive, synergistic and antagonistic effect, respectively^40^. Noticeably, the theory of CI relies on the physical, chemical, and mathematical pitfalls of the mass-action law^40^. This confirms that the use of this dose of prodigiosin/PU-H71 combination would provide favorable consequences.

The molecular mechanisms involved in the apoptotic pathway induced by HSP90α inhibition dependent on PU-H71 is not fully understood^65^. Inhibition of HSP90α offers the advantage of targeting multiple oncoproteins as well as tumour progression^22^. For example, downregulation of HIF-1α and NF-κB following the inhibition of HSP90α resulted in supressing the epithelial-mesenchymal transition (EMT), invasion, and motility of cancer cell lines^66^. In this manner, we believe that measuring the transcription and expression levels of HSP90α in MDA-MB-231 cells upon treatment with prodigiosin, PU-H71, and their combination might help explain the complex behaviour of TNBC resistance to current therapies.

Amongst all treatment groups, prodigiosin/PU-H71 combination significantly downregulated the HSP90α transcription and expression levels compared to the control state. This effect is due to the higher affinity of PU-H71 towards HSP90α combined with the ability of prodigiosin to reduce ATP production^14,25^. However, both drugs individually decreased the expression level of HSP90α but increased the transcription level. This increase in transcription level could be explained by the phenomenon of functional genetic compensation. Upregulation of related genes takes place following changes in protein levels or loss of protein function^67,68^. One of the possible explanations could be the rescue of MDA-MB-231 cancer cells by exogenous HSP90α expression, which could provide protection against PU-H71 regardless of mutations altering HSP90α conformational structure^26^. Another interpretation clarified that MDA-MB-231 TNBC cells recover the function of HSP90α by overexpressing the *HSP90AA1* locus in the presence of PU-H71. It was speculated that the abnormal asparagine at position 142 interrupts the physiological interaction between Y142 and S164, hampering the binding of PU-H71 to HSP90*α* without affecting its function and ATP hydrolysis^26,69^. Here, we show for the first time that prodigiosin has the ability to downregulate the expression level of HSP90α either individually or combined with PU-H71. Taken together, these data suggest that prodigiosin might be a promising strategy for cancer treatment, particularly in resistant tumours, highly expressing HSP90α.

The mitochondrial pathway of apoptosis occurs via the upregulation of BAX, downregulation of BCL2, mitochondrial outer membrane permeability (MOMP), cytochrome c release, and caspases activation^65^. Treatment of MDA-MB-231 cells with prodigiosin, PU-H71, and their combination upregulated the *BAX* transcription level in with a varied degree of significance. Normally, triggering mitochondrial apoptosis in cancer cell lines by both prodigiosin and PU-H71 necessitates BAX activation and downregulation of BCL2 to initiate cytochrome c release^42,65,70^. This fact, together with our findings that PU-H71 and prodigiosin increased the transcription level of *BAX* in MDA-MB-231 cells, led us to ascertain that BAX-deficient cells are resistant to treatment, provoking a weak induction of mitochondrial alterations. This observation is concordant with a reported study that has shown that BAX upregulation increases the cellular sensitivity to apoptosis and is considered therapeutically relevant. However, its downregulation confers resistance of tumour cells, leading to poor prognosis in breast cancer^53^. Our results may deduce a potential outcome for bacterial prodigiosin which may increase the *BAX* upregulation in resistant TNBC cells, increasing their sensitivity to apoptosis.

It is still unclear how apoptosis is controlled in human breast cancer cells^71^. Pertaining to BCL2, it is one of the common survival mediators and treatment resistance in most human cancers^72^. Based on previous reports, we expected to observe a decrease in the transcription level of *BCL2* by both drugs. Nevertheless, all treatment groups had an upregulatory effect except the combined therapy that did not alter the transcription level. The present finding is supported by a report in 2016 signifying that MDA-MB-231 exhibited the highest mRNA levels of *BCL2* upon treatment^71^ stating that the observed decrease in the BCL2 protein level in MDA-MB-231 represents higher levels of *BCL2* mRNA^73,74^. Another possible strong clarification is the BCL2-mediated stability by nucleolin, the *BCL2* mRNA binding protein^75^. Conversely, it was demonstrated that elevated levels of *BAX* associated with low mRNA levels of *BCL2* were correlated to the higher apoptosis rate in treated cells^19^. Our findings in agreement with another study suggest that the activity of PU-H71 is weakened by the upregulation of *BCL2*^76^.

Presently, the current combined therapy in our study was designed to block both the primary and secondary targets that might mediate resistance due to the fact that the inhibition of *Raf-1* yields good responses in TNBC^52^. Here, the transcription level of *Raf-1* increased in all treatment groups. Such increase might be explained by the parallel increase in the transcriptional levels of *HSP90α* since Raf-1 is considered as one of its potential client oncoproteins in breast cancer^77^. The increase in the transcriptional level of *Raf-1* initiated by PU-H71 was opposed by a study that elicited that the antitumour activity of PU-H71 was shown to have potent and lasting growth inhibitory effects in mouse xenografts of TNBC and depletion of oncogenes, including *Akt*, *EGFR*, and *Raf-1*. Moreover, in the same study, in Ewing sarcoma, exposure to PU-H71 resulted in depletion of critical proteins, including AKT, pERK, Raf-1, c-MYC, c-KIT, IGF1R, hTERT, and EWS-FLI^78^.

Mounting evidence speculated that sustained and overexpression of survivin is cancer specific. Survivin is an exciting tumour biomarker and a potential therapeutic target that is highly expressed in TNBC cell lines. It is linked to disease aggressiveness, clinical progression, and treatment resistance^55,79–82^. With respect to the transcription analysis, prodigiosin alone and its combination with PU-H71 did not change the survivin transcription level, opposing to PU-H71 alone that increased its levels. Interestingly, all treatment groups downregulated the expression level of survivin. It was described that survivin is involved in therapeutic modulation, which is critically regulated by interaction with prominent cell-signalling pathways including HSP90α, mTOR, BCL2, EGFR, and VEGF^83,84^. As demonstrated above, a combination of prodigiosin/PU-H71 downregulated the expression level of HSP90α. In addition, the downregulation on the expression level of survivin initiated by PU-H71 may be supported by the involvement of survivin with HSP90α to provide adaptation under cellular stress conditions by maintaining stability, folding and subcellular trafficking^83^. This also could explain the effect of PU-H71 on altering the transcription and expression levels of survivin. In agreement with another study, our study could be an important endeavour to prove that the dissociation of this ‘HSP90α-survivin complex’ leads to proteasomal degradation of survivin, mitochondrial apoptosis, and inhibits cell metastasis^85^.

Cyclin-dependent kinase-1, a protein encoded by *CDK1* gene, is a set of Ser/Thr kinase systems and is an essential factor involved in cell division, proliferation, contributing to the migration and invasion of breast cancer^56,86^. In TNBC, CDK1 was reported to be overexpressed and directly correlated with the clinicopathological features and bad prognosis^87^. For these reasons, inhibition of CDK1 is sufficient to result in a G2-M block and CDK1 which may have a role in the adjuvant treatment of TNBC^20,87,88^. Our findings reported that prodigiosin showed an unusual increase in the *CDK1* transcription level, which is discordant with a study that reported that prodigiosin inhibits the transcription level of *CDK1*^20^. Also, the prodigiosin/PU-H71combination therapy failed to downregulate the transcription level of *CDK1*. MDA-MB-231 cells could confer resistance to treatment since HSP90α exists in complex with CDK1, maintaining its activity^89^. However, PU-H71 alone, decreased marginally the *CDK1* transcription, which was reported to be in agreement with Caldas-lopes et al.^31^.

The final stage of apoptosis occurs upon cytochrome c release by cytotoxic agents, activating caspase 9, which then stimulates the activity of caspase 3, ultimately resulting in apoptosis^57,90^. In mammals, the ‘caspases’ are a highly specific class of cysteine proteases^57^ that can initiate/suppress programmed cell death via activation/deactivation of several proteins^91,92^. Treatment with prodigiosin alone and its combination with PU-H71 increased the level of *caspase 8* and *9* transcription in contrast to PU-H71, which is in agreement with Li and his colleagues in 2018^93^, stating that the proposed anticancer effects of prodigiosin is caspase-dependent. PU-H71 did not increase caspase 9 level opposing to a previous study that reported the activation of caspase 9 upon using PU-H71^94^. Noticeably, prodigiosin displayed multimodal anticancer potency as a result of caspase-dependent and -independent induction of apoptosis, activation of protein kinase pathways, and induction of cell cycle arrest^93^. Consistent with previous findings, we elucidated that prodigiosin induces mitochondrial-dependent apoptosis by the upregulation of *BAX* and *caspase 9* transcription levels. MDA-MB-231 cells that exhibit lower levels of *caspase 8* were characterised by high metastatic capacity due to a striking increase in VEGF^95^. Therefore, activating *caspase 8* gene transcription might seem good for use in cancer treatment by apoptosis through truncation of Bid (BH3 interacting-domain)^70^.

Compared with other breast cancer subtypes, EGFR is frequently mutated or upregulated in TNBC, promoting tumour progression, disease relapse, drug resistance and is a negative prognostic factor^58,96^. Normally, its activation promotes cell survival and differentiation via activation of various downstream signalling pathways, such as Ras-Raf-MEK-ERK, PI3K-AKT-mTOR, and Src-STAT3^97^. All treatment groups decreased the levels of EGFR protein expression. Our finding is in harmony with two recent studies that explained that targeting HSP90α provides the likelihood of simultaneously disrupts EGFR is one of the HSP90α client proteins and a hallmark of cancer^26,98^.

Amongst other types of breast cancers, TNBC has the most extensive vascularisation with significantly high expression levels of VEGF. This has led to postulate a specifically higher activity of antiangiogenic drugs in TNBC^99^. We examined the protein expression level of VEGF in MDA-MB-231 cell line using ELISA. Similar to EGFR, all treatment groups decreased the VEGF expression level, which might be attributed to the downregulation of HSP90α. Downstream effectors of VEGF-dependent signalling are modulated by HSP90α, and given that VEGF shares a dependence upon HSP90α, HSP90α inhibitors have the potential to target multiple levels of this pathway. HSP90α inhibition is considered as a multifaceted strategy to combat drug resistance and tumour vascularity^100^.

The PI3K-AKT-mTOR (PAM) signalling pathway, frequently overactivated in TNBC as to other subtypes, represents the main pathway responsible for cell proliferation, survival, and metabolism^60,101^. Inhibition of mTOR leads to decreased levels of HIF-1 and VEGF as reported by in-vitro studies^101^. All treatment groups decreased the expression levels of mTOR with a varied degree of significance. Prodigiosin-treated groups experience the most significant reduction as compared to control cells. In agreement with these results, Giulino-Roth and collaborators found that HSP90α inhibition targets multiple components of the PAM signalling in Burkitt’s lymphoma (BL), highlighting its importance in cancer therapy^102^. There was also a robust philosophy to investigate the association between an anti-EGFR and mTOR inhibitor to overcome the anti-EGFR resistance since EGFR is overexpressed and upregulated in almost 50% of TNBC tumours^102^.

Caspase 3 (and its cleavage CC3) is the central member of the caspase family, which is cleaved into 29- and 85-kDa fragments by PARP-1 during the early stages of apoptosis, mediating tumour repopulation in apoptotic tumour cells^103^. Recent studies have revealed the close association of caspase 3 expression and breast cancer where a disease with lower or absent apoptosis index has a bad prognosis^55,103^. The caspase 3/9 activation was associated with inhibited cell proliferation, induced apoptosis, as well as increased BAX protein expression in prostate cancer^31,104^. Prodigiosin/PU-H71 combination upregulated the level of caspase 3 protein in opposition to both drugs individually. The combination inhibits the direct binding of HSP90α to Apaf-1, facilitating the recruitment of procaspase 9, thus allowing the assembly of apoptosome and the subsequent caspase 3 activation^105^.

In-silico prediction is an ideal opportunity that creates a high interest in pharmaceutical research^106,107^. ADME properties of prodigiosin and PU-H71 were calculated to predict both physicochemically significant descriptors and pharmacokinetic properties. Generally, prodigiosin and PU-H71 could be better absorbed from the intestinal tract upon oral administration. Based on drug-likeness score, prodigiosin and PU-H71 were considered as potential therapeutic molecules and could be selected for synthesis. “Drug-likeness” is outlined as a balance between different molecular and structural attributes which determine whether or not a particular molecule is analogous to the common drugs. In a living organism, these features affect the bioavailability, transport, affinity to proteins, reactivity, toxicity, and metabolic stability of molecules. The MOLSOFT drug-likeness model score calculator is a support vector machine (SVM) classifier. Its input comprises binary chemical fingerprints (vector of 0/1, where each bit represents a particular fragment from the training set). In this model, the input molecule is converted to binary fingerprint, which is then passed to the SVM model to calculate the drug-likeness score of “decision value”. This non-linear model takes into an account the presence/absence of difference chemical fragments to calculate the score). Based on the score distribution, molecules with scores falling in a range from -2.0 to 2.0 are considered as drug-like candidates. However, molecules outside that range may potentially have some problems as drug-likes. In the present study, by looking at the score distribution among drug-like candidates, both prodigiosin and PU-H71 fall under the blue curve (drug-like molecules) (Fig. 6) with scores of -0.57 and 0.93, respectively, which demonstrates that they may be potential drug candidates^46,108^. Similarly, prodigiosin has proved as a drug-like compound according to all drug-likeness filters: The Ghose, Veber, Egan, and Muegge filters^44^. These methods were adapted from references109-112, respectively^109–112^. Also, PU-H71 has been shown to be a drug-like according to all previous filters except the Ghose methods. In computer-aided drug design (CADD), considering SA is crucial in order to select the most valuable molecules that could be synthesised and submitted for biological assessment or other tests^44^. Primarily, the SA score is based on presuming that the frequency of molecular fragments in available molecules corresponds to the ease-of-synthesis. The fragmental contribution to SA should be beneficial for common chemical moieties and unfavourable for infrequent moieties. Using the SWISSADME SA, prodigiosin and PU-H71 demonstrated SA scores of 4.29 and 3.61, respectively. The SA score is normalized to a range from 1 (very easy) to 10 (very difficult to synthesise)^44^.

The ADME properties of prodigiosin and PU-H71 were calculated to predict both physicochemically significant descriptors and pharmacokinetically significant properties. There is an inverse relationship between the molecular weight of compounds and absorption, distribution, and transportation. Such parameters decrease with the increase in molecular weight of compounds. Here, prodigiosin and PU-H71 have zero and one violation to “Lipinski’s rule of five”, respectively (Table 3). This rule defines drug-likeness for compounds, and it has the following parameters: “Mwt ≤ 500, log P ≤ 5, hydrogen bond donors ≤ 5, hydrogen bond acceptors ≤ 10”^113^. Hence, they were compliant and regarded as orally active. Prodigiosin has a molecular weight of 323.43 g/mol, number of hydrogen bond donors and acceptors was 2, and the value of MLog P was 1.58. Additionally, the Mwt of PU-H71 was 512.37 g/mol, number of hydrogen bond donors and acceptors was 6 and 2, respectively, and MLogP value was 2.12. The MLogP is used in rational drug design to assess the lipophilic potency of drugs^113^. The TPSA values for prodigiosin and PU-H71 were 53.17 Å^2^ and 125.41 Å^2^, falling in the optimal range of polarity between 20 and 130 Å^2^ as denoted by Daina et al. in 2017^44^. This can give insights concerning the polar characteristics of prodigiosin and PU-H71^44^. For toxicity classes, prodigiosin belongs to the class V, which represents molecules that may be harmful if swallowed (2000 < LD_50_ ≤ 5,000), whereas PU-H71 was found to be related to class III that comprises toxic molecules if swallowed (50 < LD_50_ ≤ 300). Collectively, these parameters demonstrated that prodigiosin and PU-H71 might be very good anticancer candidates in the future.

## Conclusions

Prodigiosin/PU-H71 combination showed potent cytotoxicity on MDA-MB-231 cells; proving to play a paramount role in interfering with key signalling pathways in TNBC. This study may contribute to the confirmation of the fact that HSP90α inhibition targets multiple components of PAM signalling highlighting its importance in cancer therapy. Interestingly, prodigiosin might be a potential anticancer agent to increase the sensitivity of breast cancer cells to apoptosis. Transcription and expression analyses suggest that the proposed anticancer effects of prodigiosin are caspase-dependent, inducing mitochondrial-dependent apoptosis by downregulation of *BCL2* and upregulation of *BAX* and *caspase 9* mRNA transcription levels.

## Recommendations

Further investigations will be interesting to explain the possible strong crosstalk between oncogenic proteins in proliferation, survival, and metastasis-related signalling pathways in MDA-MB-231 cells. Also, other investigations are needed to clarify the mechanisms by which prodigiosin and/or PU-H71 have induced cytotoxic effect and verify to what extent other biomarkers and signalling pathways were affected. We suggest the initiation of further in-vivo studies for the evaluation of antitumour effects of prodigiosin and/or PU-H71 on TNBC. More studies are recommended to elucidate whether the same findings using such drugs would be observed while dealing with different breast cancer cell lines other than MDA-MB-231 cells such as MCF-7 and T4-7D that express different genotype profiles. In-silico analyses are also advisable to examine the potential of other members of the “prodiginines” family as anticancer agents.

## Materials and methods

### Drugs under study

PU-H71 (HPLC) ≥ 98% (Sigma-Aldrich Co., USA) was prepared in a stock concentration of 10 mM using dimethyl sulfoxide (DMSO) and stored at -20 °C. Standard prodigiosin > 95% (HPLC) was purchased from Abcam PLC, UK. *S. marcescens*, a prodigiosin producer, was obtained from the culture collection of Microbial Biotechnology Laboratory, Institute of Graduate Studies and Research, Alexandria University. The strain was previously identified using VITEK 2 biochemical identification at the Faculty of Medicine, Department of Microbiology, Alexandria University. Prodigiosin was obtained from *S*. *marcescens* by extraction using a 7-L laboratory scale fermenter (CLEVER, UK) and purification as reported previously^114–118^. Purification of prodigiosin was carried out using gravity column chromatography^119^. Characterisation was performed using high-performance liquid chromatography (HPLC)^120,121^ and Fourier-Transform Infrared (FTIR) spectroscopy^114,117^.

### Production and extraction of prodigiosin

Petroleum ether HPLC grade, sodium hydroxide, hydrochloric acid, ethanol (96%), *n*-hexane, ethyl acetate, methanol, silica gel, and thin layer plates were purchased from Sigma-Aldrich Co., USA.

### Cell culture

Human MDA-MB-231 TNBC cells were purchased from the American Type Culture Collection (ATCC), USA, were cultured in Dulbecco’s Modified Eagle’s Medium (DMEM), high glucose with L-glutamine supplemented with 10% foetal bovine serum (FBS) (Biowest, Nuaillé, France), 100 U/ml penicillin, and 100 μg/ml streptomycin (Lonza Verviers Sprl, Belgium) at 37 °C in humidified air containing 5% CO_2_. Cell line was tested for mycoplasma by PCR. All experimental procedures followed the regulatory aspects with regards to the use of cell line.

### The neutral red viability assay

Drugs cytotoxicity was determined by the neutral red cytotoxicity assay^35^ where the optical density (OD) of the neutral red extract has λ_max_ at 540 nm and is directly proportional to the number of viable cells. Cells were plated at a concentration of 5,000 cells/well in a 96-well microtiter plate and were then incubated at 37 °C in 5% CO_2_ for 24 h. Culture media were replaced with 200 µl treatment media containing the drugs at different concentrations (Prodigiosin: 0.25, 0.50, 0.75, 1.00, 1.25, 1.50, 1.75, 2.00, 2.25 & 2.50 µM), (PU-H71: 10, 50, 100, 200, 250, 300 & 400 nM) followed by incubation at the same conditions for 48 h. Compusyn software version 3.0.1 was used to calculate the IC_50_ of prodigiosin and PU-H71.

### ELISA

Determination of total protein content was carried out by the Bradford assay ^122^. Total protein was extracted from 1 × 10^8^ cells for analysis by ELISA kits (human immunoassay kits) for the following: Caspase 3 (Sigma Aldrich, USA), HSP90α (Picokine Elisa, Bosterbio, USA), Survivin (Lsbio, USA), VEGF and EGFR (Invitrogen, USA), and mTOR (Abcam PLC, USA). Culture supernatants were collected for protein assay, protein levels, as determined following the manufacturer’s manual, were normalized by cell viability for each treatment. Independent experiments with three replicates for each protein were carried out, and the mean abundances from each of the experiments were pooled for statistical analysis.

### Primers and RT-qPCR

All primers used in this study were synthesised by Integrated DNA Technology Co., USA. Total RNA was extracted from 1 × 10^7^ cells by Thermo Scientific GeneJet RNA purification kit. Reverse Transcriptase (RT) reactions were done with 1 μg of total RNA by Thermo Scientific RevertAid First Strand cDNA Synthesis Kit. Gene expression was normalised by *GAPDH* gene with ∆C_T_ method^123,124^. Independent experiments with three replicates for each gene were performed, and the mean abundances from each of the experiments were pooled for statistical analysis. qPCRs were performed using Thermo Scientific Maxima Sybr Green qPCR Master Mix (2X), and 1 µM of each primer pair (Forward + Reverse) for genes (5′-3′):

*HSP90α*: F-GTGAACCTATGGGTCGTGG; R-GGGATATCCAATAAACTGAG

*Raf-1*: F-CTCCATGAAGGCTTAACAGTG; R-TGGGTTGTTATCCTGCATTCG

*Survivin*: F- GGACCACCGCATCTCTACAT; R-ACCCTTCCAGCTCCTTGAAG

*BAX*: F-GTCCAGCTCTTTAATGCCCG; R-TCCCGCCACAAAGATGGTC

*BCL2*: F-CATCAGGAAGGCTAGAGTTACC; R-CAGACATTCGGAGACCACAC

*Caspase 9*: F-GTGGACATTGGTTCTGGAGGAT; R-CGCAACTTCTCACAGTCGATG

*CDK1*: F-GTAGTAACACTCTGGTACAG’ R-CAATTTCTGAATCCCCATGG

*Caspase 8*: F- CCTGGGTGCGTCCACTTT; R-CAAGGTTCAAGTGACCAACTCAAG

*GAPDH*: F-GCACAACAGGAAGAGAGAGACC; R-AGGGGAGATTCAGTGTGGTG.

### In-silico analyses

Prodigiosin and PU-H71 were subjected to drug-likeness prediction and ADME properties using MOLSOFT’s chemical fingerprints and SWISSADME, using canonical SMILES as input^44,46,108^. Furthermore, lethal dose 50 (LD_50_) was determined to predict oral toxicities of small molecules in rodents (described as LD_50_ in mg/kg body weight). Prediction of toxicity for prodigiosin and PU-H71 was performed using PROTOX-II, a webserver for toxicity prediction of chemicals^45^. Toxicity classes for both drugs were defined according to the globally harmonised system of classification of labelling of chemicals (GHS).

### Statistical analysis

All results were expressed as mean ± standard error of the mean (s.e.m.). Multiple comparisons were done using one-way analysis of variance (ANOVA) followed by the posthoc test and the *P*-value < 0.05 was accepted as the level of significance. All statistical tests and Figures were carried out using GRAPHPAD PRISM version 6 **(**GRAPHPAD software, USA).

## Data Availability

The datasets generated during and/or analysed during the current study are included in this article.
